# The N2 dilemma in stage III NSCLC: toward a quantitative framework for trial design

**DOI:** 10.3389/fonc.2026.1866606

**Published:** 2026-10-09

**Authors:** Luca Bertolaccini, Monica Casiraghi, Antonio Mazzella, Giorgio Lo Iacono, Lorenzo Spaggiari

**Affiliations:** 1 Department of Oncology and Hemato-Oncology, University of Milan, Milan, Italy; 2 Department of Thoracic Surgery, European Institute of Oncology IRCCS, Milan, Italy

**Keywords:** clinical trial design, lung cancer, N2 disease, resectability, stage III NSCLC

Stage III non-small-cell lung cancer (NSCLC) represents one of the most clinically and methodologically challenging entities in thoracic oncology, where anatomical staging, biological behavior, and treatment strategies intersect without full alignment. Despite substantial advances in multimodality therapy, including chemo-immunotherapy and refined surgical techniques, the field continues to face a fundamental limitation: the lack of a reproducible and universally accepted definition of mediastinal nodal disease, particularly N2 involvement. This misalignment is not merely conceptual but has direct consequences on trial validity and interpretability. Importantly, two distinct but interconnected sources of heterogeneity should be recognized. The first is biological heterogeneity, reflecting intrinsic differences in tumor biology, including molecular alterations, immune microenvironment, and treatment responsiveness. The second is measurement heterogeneity, resulting from variability in imaging acquisition, nodal staging techniques, interpretation, and reporting across institutions. While biological heterogeneity requires integration of biomarkers, measurement heterogeneity demands standardized quantitative assessment. The framework proposed here is intended to address both dimensions.

The recent multisocietal effort led by the European Organization for Research and Treatment of Cancer (EORTC) represents a significant attempt to standardize the definition of technical resectability in stage III NSCLC (1). This initiative is timely and necessary, given the increasing number of clinical trials exploring perioperative and multimodal strategies. However, while the consensus successfully frames resectability as a multidisciplinary, context-dependent decision, it also exposes, rather than resolves, the ambiguity surrounding N2 disease, which remains the central determinant of treatment allocation. Specifically, important aspects remain only partially operationalized, including the definition of bulky nodal disease, the distinction between single- and multi-station involvement, and the incorporation of functional imaging into resectability assessment.

In contrast, contiguous and non-contiguous disease describe direct versus anatomically discontinuous mediastinal nodal spread. These unresolved issues inevitably leave room for interinstitutional variability. In this context, the present perspective should be interpreted as a complementary, operational extension of the EORTC framework, aimed at enhancing its applicability in clinical trial design rather than challenging its conceptual foundation.

Indeed, N2 disease is widely recognized as a biologically and clinically heterogeneous condition, encompassing a spectrum of scenarios that differ substantially in prognosis and therapeutic implications (2). The current classification into single-station, multi-station, bulky, and invasive disease reflects an attempt to capture this heterogeneity. Yet these categories lack precise, reproducible definitions. In particular, the concept of “bulky” N2 disease remains inconsistently defined, often relying on arbitrary size thresholds that have not been prospectively validated against biological aggressiveness, surgical complexity, or patient outcomes. Throughout this article, bulky disease refers to nodal disease with an extensive tumor volume likely to compromise complete surgical resection.

This definitional imprecision extends beyond semantics and introduces important methodological challenges for trial validity. Eligibility criteria based on loosely defined nodal characteristics inevitably introduce misclassification bias, reduce inter-study comparability, and limit the external validity of trial results. In multicenter settings, variability in imaging interpretation, staging techniques, and institutional expertise further amplifies this problem, undermining the very objective of harmonization.

Moreover, the persistent uncertainty surrounding multi-station N2 disease underscores the limitations of the current categorical approach. Divergences between expert opinion, survey responses, and real-world case assessments suggest that the existing framework is insufficiently granular (3). As long as multi-station involvement is treated as a binary variable rather than a quantitative, spatial continuum, its integration into trial design will remain problematic.

The reliance on multidisciplinary team (MDT) discussion as a compensatory mechanism, although clinically appropriate, introduces an additional layer of variability. MDT decisions are inherently influenced by local expertise, surgical philosophy, and institutional resources. For example, a patient with single-station, borderline bulky N2 disease on imaging may be considered resectable in a high-volume surgical center, while being directed toward definitive chemoradiotherapy in another institution with different expertise or thresholds. While this flexibility is essential in clinical practice, it conflicts with the need for reproducibility and standardization in research contexts. A framework that depends on subjective consensus cannot serve as a reproducible or auditable foundation for trial eligibility criteria. Rather than replacing multidisciplinary decision-making, quantitative tools should provide standardized, reproducible inputs to support MDT deliberations while preserving expert clinical judgment. Taken together, these limitations expose a structural inconsistency between clinical decision-making and trial design.

These considerations suggest that the current paradigm for defining N2 disease is reaching its limits. A shift toward a more quantitative and biologically informed classification is needed. A pragmatic way forward would be to operationalise N2 disease using a composite, trial-ready framework integrating: (i) nodal burden (number of stations and absolute number of involved nodes), (ii) volumetric nodal assessment rather than diameter-based thresholds, (iii) anatomical distribution (contiguous vs non-contiguous spread), and (iv) biological activity (metabolic burden on PET and, where available, molecular or circulating biomarkers). Candidate biomarkers may include circulating tumor DNA, PD-L1 expression, or other validated molecular markers, with the caveat that their availability currently varies among institutions. At this stage, this framework should be regarded as a conceptual roadmap rather than a finalized prediction model. Each of these dimensions could be predefined using standardized thresholds and combined into a composite score to allow continuous or ordinal stratification of N2 disease within trial eligibility criteria. Although volumetric assessment presents practical challenges, including segmentation variability, software availability and training requirements, it offers a more faithful representation of complex nodal morphology than unidimensional measurements. Diameter-based assessment may therefore remain a pragmatic minimum standard where volumetric analysis is not feasible. Such a multidimensional model could be translated into a continuous or semi-continuous score, allowing stratification rather than binary categorization and improving both internal validity and cross-trial comparability. Integration of advanced imaging, radiomics, and molecular profiling may further enhance staging precision (4). Such an approach would align with emerging standards in prediction model development and reporting, including TRIPOD-AI. It could be prospectively evaluated for discrimination, calibration, and clinical utility within trial populations (5). Future development of such a model should follow established guidance for prediction models, including transparent handling of missing data, assessment of both discrimination and calibration, external validation, and predefined reporting standards.

Prospective validation should initially be performed in multicenter cohorts with centralized imaging review, predefined endpoints, external validation, and assessment of discrimination, calibration, and clinical utility before integration into randomized clinical trials.

The EORTC consensus should therefore be interpreted not as a definitive solution, but as a necessary transitional framework that highlights the boundaries of the current categorical paradigm. Its true value lies in identifying the gaps that must be addressed to achieve meaningful standardization. From a practical perspective, successful implementation will require standardized imaging protocols, accessible software for quantitative analysis, clinician training, and the progressive incorporation of molecular biomarkers based on local resources. Ultimately, although continuous modeling may provide greater biological and statistical granularity, translation into a limited number of clinically meaningful categories is likely to represent the most pragmatic strategy for future clinical trial design and implementation.

Until a reproducible, biologically grounded definition of N2 disease is established, stage III NSCLC will remain characterized by variability rather than uniformity. In this context, the priority is no longer to refine categorical definitions, but to establish a measurable, reproducible, and trial-ready framework that supports consistent patient selection and valid cross-trial comparisons.
